# Supplementary material for: The interactome of intact mitochondria by cross-linking mass spectrometry provides evidence for coexisting respiratory supercomplexes
Source: Mol Cell Proteomics. 2017 Dec 8;17(2):216–32. doi: 10.1074/mcp.RA117.000470 (PMC5795388; doi:10.1074/mcp.RA117.000470)
Supplement: Supplemental Data [file supp_RA117.000470_133922_0_supp_23978_fzffwf.zip › spectra_annotation/mito_spectra_annotation/23-1-14-1-4-1.pdf]

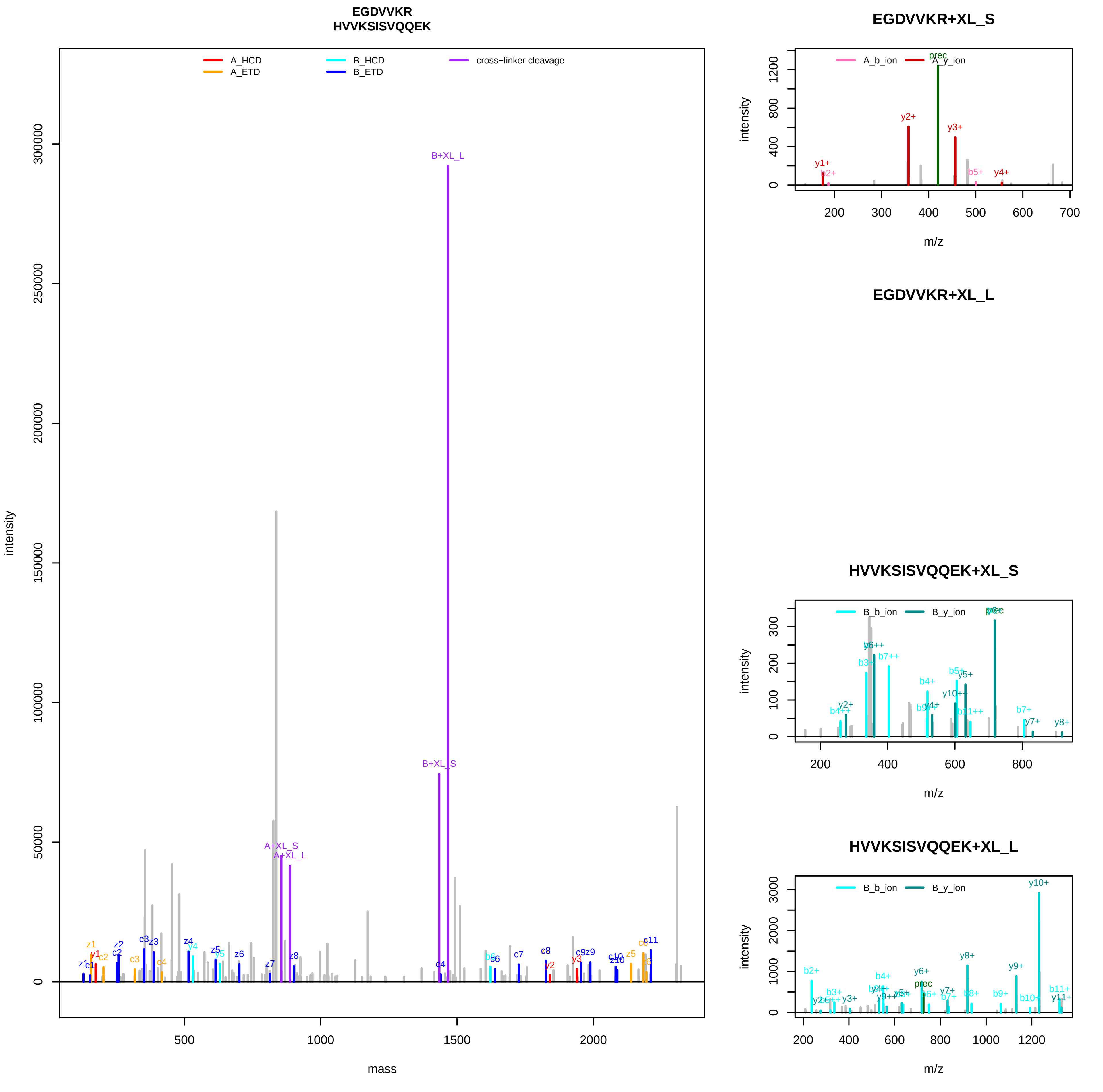

EGDVVKR
HVVKSISVQQEK
A_HCD
A_ETD
B_HCD
B_ETD
cross−linker cleavage
B+XL_L
B+XL_S
A+XL_S
A+XL_L
intensity
mass
EGDVVKR+XL_S
A_b_ion
A_y_ion
prec
y1+
b2+
y2+
y3+
b5+
y4+
m/z
EGDVVKR+XL_L
HVVKSISVQQEK+XL_S
B_b_ion
B_y_ion
y6++
b7++
b3+
b4+
b5+
y5+
y10++
y2+
b4++
y4+
b11++
b7+
y7+
y8+
HVVKSISVQQEK+XL_L
y10+
y8+
y9+
y6+
b2+
b4+
y4+
prec
y3+
y7+
b11+
y11+
